# Neuropsychological assessment in vascular cognitive impairment: A call to lay the quest for the best test to rest

**DOI:** 10.1016/j.cccb.2024.100219

**Published:** 2024-03-23

**Authors:** Geert Jan Biessels

**Affiliations:** Department of Neurology, UMC Utrecht Brain Center, University Medical Center Utrecht, Utrecht, The Netherlands

**Keywords:** Clinical trials, Cognitive testing, Differential diagnosis, Post-stroke cognitive impairment, Vacsular cognitive impairment, Vascular dementia

A recent issue of the Journal included two elegant papers on cognitive testing in the context of Vascular Cognitive Impairment (VCI) [1,2]. The first paper considered “dementia-specific variance in executive measures”, a latent variable capturing the variance in performance on tests of executive function that is empirically related to impairments in instrumental activities of daily living (IADL) [1]. The resulting construct (referred to as “δ” for dementia) is considered to offer a dementia-specific cognitive phenotype, that was previously shown to be related to dementia severity. Despite the attractive concept of thus integrating cognitive test performance and IADL, the authors observed that MRI manifestations of ischemic small vessel disease (SVD) had few independent effects on this executive measure in models that also included general intelligence. The other paper also addressed interrelations between cognitive test performance and intelligence [2]. It first summarized earlier studies reporting that cognitive reserve -defined as intellectual abilities attained by young adulthood- increases resilience against pathologies leading to dementia in late life. It also reviewed how measures of intelligence can be derived from cognitive testing, by assessing the shared variance of all tests in a battery, also referred to as “the Spearman's g”. The author made the point that constructs such as δ and g are essentially ‘blind’ to dementia etiology, with weak associations with etiological biomarkers and thus with a limited contribution to the differential diagnosis. She therefore stated that classical neuropsychological approaches based on patterns of cognitive test performances remain fundamental for differential diagnosis.

For me these papers also exemplify challenges in cognitive testing in VCI. Although there has been great progress both in cognitive neuropsychology, including development of novel (computerized) test and expanding insights in fundamental cognitive processes, and in diagnostic biomarkers for VCI, in particular using MRI, there is still no widespread consensus on how cognitive testing in VCI should best be performed. While standards for cognitive testing in VCI have been advocated [3,4], over the years I have attended quite a few debates at conferences and symposia on how to best test cognition in VCI. My expectations are that this issue will remain unresolved, because this question has so many dimensions to it that it likely does not have a single answer. This is primarily due to the fact that VCI itself is such a heterogenous construct [5]. There are various underlying vascular pathologies, that can affect different parts of the brain, either as a single insult, or in the form of progressive disease, often with various comorbidities, all in all with a highly varied cognitive phenotype, that can be static, deteriorate over time, or even improve. In addition, perspectives on “what's best” in terms of cognitive testing very much lay in the eyes of the beholder, which can be neuropsychologists, but also clinical practitioners, neuroscientists, and clearly also patients. As I am not a neuropsychologist myself, I will herein address this issue from a user perspective, as a clinician and VCI researcher.

Let's start with the perspective of diagnosing VCI in clinical practice. Typically, the starting point of the diagnostic journey are a patient's complaints. Unless cognitive complaints directly follow a stroke, one can generally not know for sure that “V” is the actual cause of the “CI”, just based on the patient's history and complaints alone. There often is a differential diagnosis, including Alzheimer's disease (AD) or Parkinsonian disorders, among others. Hence, because cognitive testing is often performed before the primary diagnosis is established, it is likely most effective to use a generic protocol for the diagnostic work-up in daily practice, assessing the main cognitive domains affected by the commonest dementia subtypes, rather than to use a dedicated protocol fine-tuned to one particular dementia subtype such as VCI. Clearly, specific add-ons may then be added to this generic protocol, directed by the patient's complaints.

Another point of debate is how well cognitive profile guides the etiological differential diagnosis. The prevailing view has long been that the pattern of cognitive deficits points at specific causes. Thus, deficits in frontal-executive function are conventionally considered to be typical for VCI, whereas prominent episodic memory deficits are more readily attributed to AD [6]. These conventions have emerged from a long clinical tradition. Just 20 years ago, the etiological diagnosis of dementia could only be ascertained definitely after death, at autopsy. During life one could only establish syndromal diagnoses, based on the constellation of a patient's symptoms. Because syndromal diagnostic criteria were carefully crafted from years of empirical clinical observations, they are often right and we should not diminish this body of knowledge. Yet, there is also widespread awareness that syndromal diagnoses lack sensitivity and specificity. This is particularly the case for a condition that is so inherently heterogenous as VCI. Take for example a lacunar infarct in the thalamus due to arteriolosclerosis. This form of SVD leads to widespread changes in the small perforating arteries. Which artery eventually occludes is essentially a stochastic event, resulting in a infarct location at a random position. Because the functional anatomy of the thalamus is highly complex, infarcts at different locations can result in quite different cognitive profiles [7], despite sharing the exact same underlying small vessel etiology. Along these lines, it has also been noted that other forms of SVD, including white matter hyperintensities, may relate to cognitive deficits on different domains, including memory [8,9]. Moreover, the fact that vascular injury commonly contributes to cognitive deficits in the context of mixed pathology, particularly in older people, further complicates the reliance on cognitive profiles as a differential diagnostic tool for etiology without corroborative support from other sources of diagnostic information. My expectations are that with the increasing availability of diagnostic biomarkers, for vascular injury at present predominantly imaging, preferably MRI [10], but for AD also increasingly fluid biomarkers, also from blood [11], we will rely less and less on the cognitive profile to reach what is essentially a syndromal etiological differential diagnosis, but move towards biological definitions of the different causes of dementia [12]. In this light, evaluation of how a particular cognitive tests differentiates between VCI and AD, as we have often seen reported in the literature, may at some point even become obsolete. Clearly this does not make cognitive testing less relevant for clinical diagnosis. Where biomarkers can inform on etiologies, they generally provide little insight in the actual functional status of a patient. Cognitive assessment will therefore remain a cornerstone in the evaluation of people with cognitive complaints, complementary to biomarker findings. It substantiates the presence, nature and severity of underlying cognitive deficits, supports classification into stages of cognitive dysfunction, and provides insight into an individual's cognitive strengths and weaknesses [13]. This may help patients and their caregivers to better understand and deal with the deficits and can also guide dedicated training or support programs.

For the VCI research perspective, I will focus on cognitive testing as outcome measure in clinical trials. This is important, because drug treatment for SVD that goes beyond conventional vascular risk factor management is urgently needed. Recently, the Framework for Clinical Trials in Cerebral Small Vessel Disease (FINESSE) provided guidance on trial design from experts based on a structured Delphi consensus process [14]. One key issue that was identified is that currently used cognitive tests have low sensitivity to change over time in patients with SVD. This likely relates both to the selection of patients in previous trials, who overall showed little decline, and to the features of the tests themselves [14]. One may argue, however, if developing new tests with higher sensitivity is really the way forward. In the end, drug trials, particularly definite phase 3 studies, should have clinically meaningful outcomes. Such outcomes, albeit heterogenous in nature, are well established for SVD [15], although they may manifest at a slow pace. In this light it is relevant to consider developments from the AD field, where we have seen many drug trials over the past decades. There, also in response to results of positive trials, the field tries to decide on the smallest change in an outcome that constitutes a clinically meaningful treatment effect (i.e. the minimum clinically important difference [MCID]) [16]. This is reflected, for example, in the debate on the results of the “Clarity AD trial” of lecanemab, a humanized monoclonal antibody that binds with high affinity to soluble amyloid-beta. Lecanemab reduced markers of amyloid in patients with early AD and resulted in moderately less decline on measures of cognition and function than placebo at 18 months [17]. The debate centers on the benefit/risk balance of the modest effect of lecanemab on cognition against the risk of quite substantial adverse events, as well as on costs. It is also of interest to consider other functional outcome measures that are already widely used in the field of cerebrovascular disease. In stroke trials the modified Rankin scale is almost considered a gold standard [18,19]. Currently, few people raise an eyebrow about using such a crude and insensitive test in a condition with such heterogeneous functional consequences as stroke, likely because of the clinical relevance of this outcome measure. Importantly, constructs like “δ”, as presented by Royall et all [1] are of interest also in this setting. Creating latent variables capturing the variance in cognitive performance that is empirically related to the outcome (e.g. IADL) or even the exposure or disease process of interest could make cognitive testing in drug trials in VCI more impactful.

So do we really need new more sensitive cognitive tests for trials in VCI? I guess we primarily need an open discussion on what we actually intend to measure, create efficient testing procedures, including computerized cognitive assessment, and keep an open eye for constructs that capture meaningful variance in cognitive performance or data clustering approaches that help to identify different clinical subtypes. First and foremost, we really need drugs that effectively engage key processes in the SVD disease pathway and means to select the right patients for such drugs. The ongoing debate on what cognitive tests should be used may even deter pharma from entering the field, for lack of consensus on how to design a trial. Hence, my – possibly slightly provocative - take on this would be: “please abandon the quest for the best test, lest the VCI field might go west”.

## CRediT authorship contribution statement

**Geert Jan Biessels:** Writing – review & editing, Writing – original draft, Conceptualization.

## Declaration of competing interest

No conflicts of interest.
